# Characteristics of gastric cancers developed more than 10 years after eradication of *Helicobacter pylori*

**DOI:** 10.1097/MD.0000000000040492

**Published:** 2024-11-15

**Authors:** Akiko Sasaki, Chikamasa Ichita, Chihiro Sumida, Takashi Nishino, Miki Nagayama, Jun Kawachi, Yuma Suno, Takaaki Murata, Wataru Naito, Nobutake Yamamichi

**Affiliations:** a Department of Gastroenterology, Medicine Center, Shonan Kamakura General Hospital, Kamakura City, Kanagawa, Japan; b Department of General Surgery, Shonan Kamakura General Hospital, Kamakura City, Kanagawa, Japan; c Department of Diagnostic Pathology, Shonan Kamakura General Hospital, Kamakura City, Kanagawa, Japan; d Center for Epidemiology and Preventive Medicine, Graduate School of Medicine, The University of Tokyo, Tokyo, Japan.

**Keywords:** chronic inflammation, eradication, gastric cancer, *Helicobacter pylori*, surveillance interval

## Abstract

*Helicobacter pylori* (*H pylori*) eradication is expected to effectively prevent gastric cancer (GC). However, GC cases may occur even longer than 10 years after *H pylori* eradication (L10AE). Moreover, the associated factors and characteristics are unknown. In this retrospective, single-center study conducted between 2017 and 2022, patients with GC diagnosed after *H pylori* eradication were enrolled and categorized into groups according to whether they were shorter than 10 years after *H pylori* eradication (S10AE) or L10AE. Patients were also categorized according to the depth of cancer invasion. Clinical data, pathological data, and risk factors were analyzed using logistic regression. Clinicopathological characteristics of GC diagnosed at L10AE and those invading the submucosal tissue or deeper (SMD) were investigated. In total, 202 cases of GC occurring after *H pylori* eradication were identified. Comparison of 158 S10AE and 44 L10AE GC cases revealed a significantly longer surveillance interval (SI) in L10AE cases (median: 2.0 vs 1.0 years, *P* = .01). Comparison of 150 intramucosal and 52 SMD GC cases revealed that L10AE GC cases were significantly more frequent amongst the SMD cases (18.0% vs 32.7%, *P* = .03). Pathologically, undifferentiated and mixed types were significantly more frequent in GC cases with SMD invasion (*P* < .001). Multivariate analysis revealed that L10AE was significantly related to GC cases with SMD invasion (odds ratio, 2.45; 95% confidence interval, 1.15–5.11; *P* = .019). SI was significantly longer in GC that developed at L10AE than at S10AE. In addition, GC with SMD invasion was more frequently detected in L10AE than in S10AE. Our data indicated that SI should not be groundlessly extended in patients at L10AE.

## 1. Introduction

Chronic inflammation due to *Helicobacter pylori* (*H pylori*) infection is the most common cause of gastric cancer (GC),^[1,2]^ and eradicating *H pylori* is expected to effectively prevent it.^[3,4]^ Several studies have previously shown that GC is observed even after the eradication of *H pylori*,^[5]^ which is partly explained by the persistence of abnormally accelerated DNA methylation.^[6]^ Despite annual surveillance, GC has occurred longer than 10 years after eradication of *H pylori* (L10AE). Therefore, continued endoscopic surveillance is recommended.^[7]^ However, the associated factors and characteristics of GC that develop at L10AE have not been well evaluated. Therefore, this study aimed to identify the factors associated with GC development at L10AE and investigate the clinicopathological differences between GC cases that develop at shorter than 10 years after eradication of *H pylori* (S10AE) and those that develop at L10AE.

## 2. Materials and methods

### 2.1. Study design

This was a single-center, retrospective, observational study of patients with GC after *H pylori* eradication.

### 2.2. Setting

Eligible patients were enrolled from January 2017 to December 2022 at Shonan Kamakura General Hospital, Kamakura, Japan. We included patients with primary GC, who were aged ≥ 20 years and diagnosed more than 1 year after *H pylori* eradication. All procedures were performed following the ethical standards established by the 1964 Declaration of Helsinki and its later amendments. The Institutional Review Board of Mirai Iryo Research Center Inc., Shonan Kamakura General Hospital approved this study (TGE01329-024) and waived the requirement for informed consent; instead, patients were allowed to opt out of the study.

Patients meeting the following criteria were excluded: (1) active *H pylori* infection, (2) negative for *H pylori*, without previous eradication, (3) unknown status of *H pylori* infection, (4) failure in eradication of *H pylori*, (5) history of gastrectomy, and (6) undergoing chemotherapy for the treatment of other cancers. In some cases, eradication treatment or surveillance was carried out in other hospitals or clinics. Cases where the decision of successful eradication had not been fully confirmed were excluded from the study. On the other hand, based on the concept of intention-to-treat analysis, we did not exclude the subjects when they did not undergo endoscopic surveillance.

Eligible patients were first categorized into 2 groups according to a GC diagnosis shorter or longer than 10 years after the successful eradication of *H pylori* (S10AE and L10AE), that is according to the period during which the cancer manifested.^[8]^ Second, the patients were categorized into 2 groups according to whether the depth of cancer invasion was within the mucosal tissue or the submucosal or deeper tissue (SMD). Third, the GC cases with SMD were categorized into 2 groups according to whether they were diagnosed at S10AE or L10AE.

### 2.3. Study variables

All patients’ clinical and pathological data were retrospectively collected. Clinical data included age, sex, date of *H pylori* eradication, status of atrophic gastritis, past medical history of GC, and surveillance interval (SI) from the last screening endoscopy. GC pathological data included the site, size, macroscopic classification, pathological findings, stage, and treatment.

For patients who had synchronous lesions, we selected the main tumor according to its characteristics, including tumor depth, histologic classification (undifferentiated type), and size.^[9]^ Concerning metachronous GC within the observation period, only the lesion diagnosed first was chosen.

GC after *H pylori* eradication was defined as follows^[10]^: (1) GC detected more than 1 year after successful eradication, (2) confirmed by serum antibody (E-plate, Eiken Chemical Co., Ltd., Tokyo, Japan), urea breath test (UBIT, Otsuka Chemical Co., Ltd., Tokushima, Japan), or stool antigen (Wakamoto Pharmaceutical Co., Ltd., Tokyo, Japan), and (3) endoscopic-atrophy. Gastric mucosal atrophy was evaluated according to the endoscopic-atrophy-border scale described by Kimura and Takemoto at the time of cancer detection. This correlates with the results of histological evaluation,^[11]^ and is classified into the following 2 levels: closed type (C-1, C-2, and C-3) and open type (O-1, O-2, O-3, and O-p).

All patients underwent endoscopy before treatment at our hospital, and a histological diagnosis was made by cancer biopsy. The final examination before the diagnosis of GC after the eradication was upper gastrointestinal endoscopy or upper gastrointestinal fluoroscopy. The detailed classification items of gastritis, such as intestinal metaplasia, and the histological evaluation of atrophy were not performed. The images were confirmed and reviewed by at least 2 specialists from the Japanese Society of Gastrointestinal Endoscopy during GC diagnosis and, in possible cases, at the final examination before GC diagnosis.

Furthermore, GC was categorized into 2 types according to the Kanno–Nakamura classification^[12]^: differentiated carcinoma, well-differentiated adenocarcinoma (tub1), moderately differentiated adenocarcinoma (tub2), papillary adenocarcinoma (pap) and undifferentiated carcinoma; and carcinoma with a predominance of poorly differentiated adenocarcinoma (por) and signet-ring cell carcinoma (sig).^[13]^ In terms of inoperable stage-IV advanced GC cases, patients with advanced carcinoma clearly diagnosed using endoscopic findings and computed tomography images were considered to have advanced carcinoma deeper than the intrinsic muscularis propria.

### 2.4. Statistical analysis

Continuous variables are presented as medians and ranges and were analyzed using the Mann–Whitney *U* test for nonparametric data. For the analysis of the period, we converted the duration into years for easier calculation, such as converting 17 months into 1.4 years. The other categorical variables are expressed as numbers and percentages and were analyzed using Fisher exact test. A *P*-value of <.05 was considered statistically significant. Multivariate analyses were performed for the clinical background related to the carcinogenesis of GC, including age, sex, atrophic state of gastritis, past medical history of GC, and period from eradication of *H pylori*. All statistical analyses were performed using EZR ver.1.55,^[14]^ which is a package for R statistical software (https://www.r-project.org/) (R Software for Statistical Computing, Vienna, Austria). For each of the listed study variables, the background factors that were significant for 2 groups and clinically characteristic items, including age, sex, state of atrophic gastritis,^[7]^ past medical history of GC,^[15]^ and the periods from the eradication of *H pylori* were used to evaluate the data using logistic regression.

## 3. Results

### 3.1. GC developed at longer than 10 years after *H pylori* eradication had a longer SI and was more advanced

Overall, 202 cases of GC after eradication of *H pylori* were detected out of a total of 960 cases of GC diagnosed at our hospital. Among them, there were 4 cases diagnosed by biopsy and were treated with chemotherapy, and 1 case that received the best supportive care without chemotherapy.

**Figure 1.**
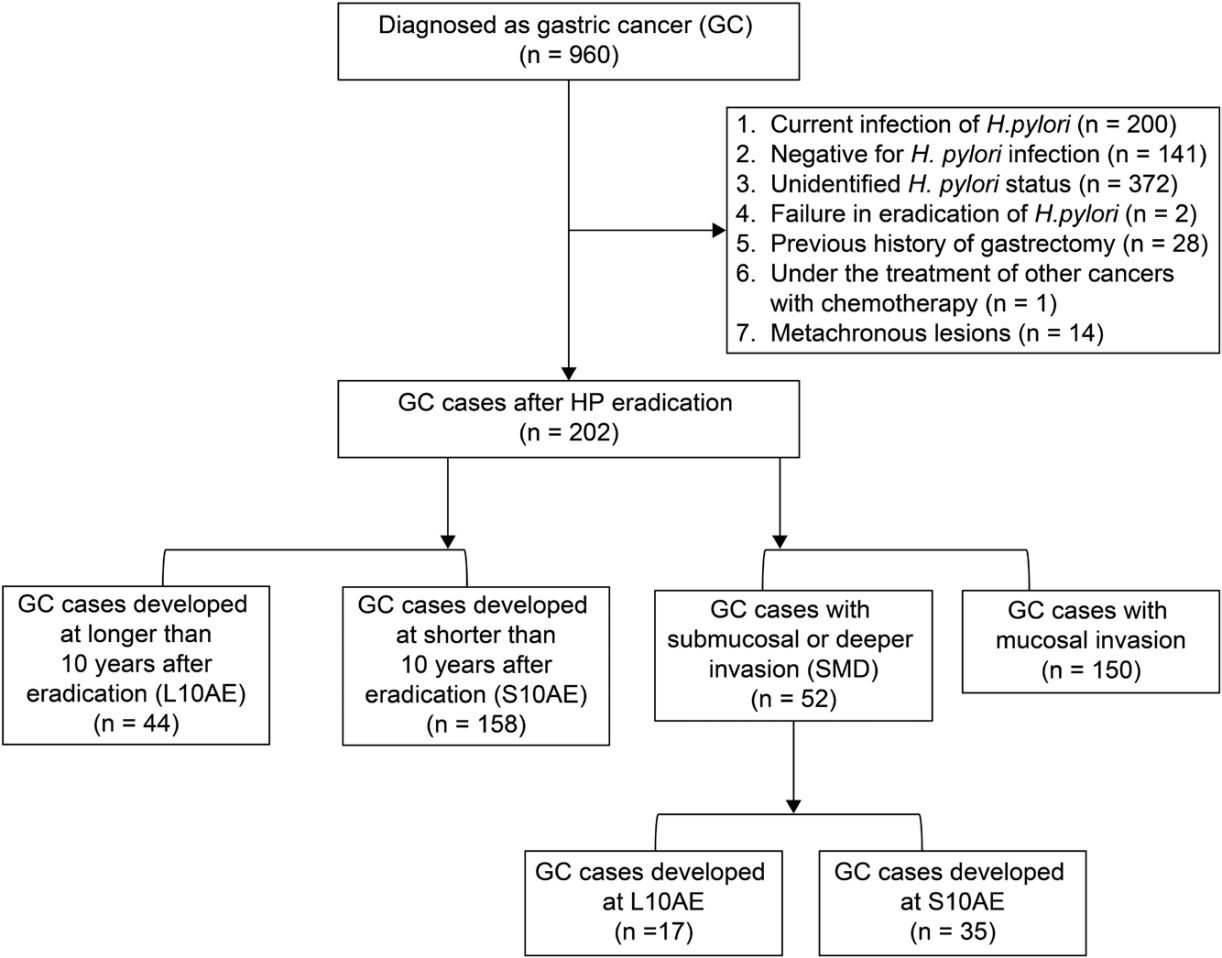
Study flowchart.

Initially, they were divided into 2 groups according to the time from eradication of *H pylori* to the time of diagnosis (158 and 44 cases of GC developed at S10AE and L10AE, respectively) (Fig. 1). Regarding sex, age, and past medical history of GC, there were no statistically significant differences between S10AE and L10AE GC cases. Atrophy of gastric mucosa (atrophic gastritis) tended to be milder in the GC cases at L10AE. Although there were no significant differences in tumor site or histology, more advanced cancers were detected in GC developed at L10AE. In addition, GC developed at L10AE had a significantly longer SI than that developed at S10AE (median: 2.0 vs 1.0 years, *P* = .01) (Table 1).

**Table 1:** Univariate analysis for the clinical background and pathological characteristics of study patients categorized by period from *Helicobacter pylori* eradication.

		More than 10 years (n = 44)	<10 years (n = 158)	*P*-value
Period from eradication, years, median (min, max)		10.0 [10.0, 30.0]	4.0 [1.0, 9.0]	
Age, years, median (min, max)		74.5 [54.0, 89.0]	75.0 [44.0, 93.0]	.76
Sex, n (%)	Female	9 (20.5)	48 (30.4)	.26
	Male	35 (79.5)	110 (69.6)	
Last SI, years, median (IQR)		2.0 [1.0, 3.0]	1.0 [1.0, 2.0]	.01
Atrophic gastritis, n (%)	Closed type	11 (25.0)	22 (13.9)	.11
	Open type	33 (75.0)	136 (86.1)	
PMH of GC, n (%)	Yes	5 (11.4)	34 (21.5)	.19
UML, n (%)	U	5 (11.4)	20 (12.7)	.84
	M	16 (36.4)	64 (40.5)	
	L	23 (52.3)	74 (46.8)	
Pathology, n (%)	Diff.	34 (77.3)	131 (82.0)	.52
	Mixed	6 (13.6)	14 (8.9)	
	Undiff.	4 (9.1)	13 (8.2)	
pT, n (%)	m	27 (61.4)	123 (77.8)	.033
	SMD	17 (38.6)	35 (22.2)	
Stage, n (%)	IA	34 (77.3)	144 (91.1)	.017
	IB-IV	10 (22.7)	14 (8.8)	
Treatment, n (%)	ESD	33 (75.0)	134 (84.8)	.18
	Surgery or chemotherapy	11 (25.0)	24 (15.2)	

diff. = differentiated adenocarcinoma, ESD = endoscopic submucosal dissection, GC = gastric cancer, IQR = interquartile range, L = lower third of the stomach, last SI = interval from last surveillance, m = intramucosal lesion, M = middle third of the stomach, PMH = past medical history, pT = depth of tumor invasion, SMD = submucosal or deeper invasion, U = upper third of the stomach, undiff. = undifferentiated adenocarcinoma.

### 3.2. GC with submucosal tissue or deeper invasion was predominantly undifferentiated and of mixed type

Next, the patients were divided into 2 groups according to the depth of cancer invasion: 150 intramucosal GC cases and 52 SMD invasion GC cases (Fig. 1). Regarding sex, age, severity of atrophic gastritis, and past medical history of GC, there were no statistically significant differences between the 2 groups (Table 2). The GC with SMD invasion was detected more frequently in L10AE cases with statistical difference (32.7% vs 18.0%, *P* = .03). In terms of pathology, undifferentiated and mixed type GC were more frequently observed in the invasive cancers with statistical difference (undifferentiated: 21.1% vs 3.3%, mixed: 26.9% vs 4.0%, *P* < .001). In addition, GC with SMD invasion tended to be located at the upper third of the stomach region. Multivariate analysis of the clinical characteristics revealed that the GC development at L10AE was a significant risk factor for SMD invasion (odds ratio, 2.45; 95% confidence interval, 1.15–5.11; *P* = .019) (Table 3).

**Table 2:** Univariate analysis for the clinical and pathological background of study patients categorized by the depth of cancer invasion.

		GC cases with SMD (n = 52)	GC cases with mucosal invasion (n = 150)	*P*-value
Age, years, median (min, max)		73.0 (50.0, 93.0)	75.0 (44.0, 90.0)	.64
Sex, n (%)	Female	16 (30.8)	41 (27.3)	.72
	Male	36 (69.8)	109 (72.7)	
Atrophic gastritis, n (%)	Closed type	6 (11.5)	27 (18.0)	.38
	Open type	46 (88.5)	123 (82.0)	
PMH of GC, n (%)	Yes	7 (13.5)	32 (21.3)	.31
	No	45 (86.5)	118 (78.7)	
Period from HP eradication, n (%)	More than 10 years	17 (32.7)	27 (18.0)	.033
	<10 years	35 (67.3)	123 (82.0)	
Last SI, years, median (IQR)		3.0 (1.0, 5.0)	1.0 (1.0, 2.0)	<.001
UML, n (%)	U	10 (19.2)	15 (10.0)	.17
	M	21 (40.4)	59 (39.3)	
	L	21 (40.4)	76 (50.7)	
Size, mm, median (IQR)		30 (17.3, 45.0)	13 (7.0, 20.0)	<.001
Macroscopic type, n (%)	Type 0-I, 0-IIa (early-stage GC)	4 (7.7)	39 (26.0)	<.001
	Type 0-IIb, 0-IIc (early-stage GC) Type 1-5 (advanced-stage GC)	28 (53.8) 20 (38.5)	111 (74.0) 0 (0)	
Pathology, n (%)	Diff.	26 (50.0)	139 (92.7)	<.001
	Mixed	14 (26.9)	6 (4.0)	
	Undiff.	12 (23.1)	5 (3.3)	
Stage, n (%)	IA	28 (53.8)	150 (100.0)	<.001
	IB-IV	24 (46.2)	0 (0.0)	
Treatment, n (%)	ESD	22 (41.5)	145 (96.7)	<.001
	Surgery or chemotherapy	31 (58.5)	5 (3.3)	

diff. = differentiated adenocarcinoma, ESD = endoscopic submucosal dissection, GC = gastric cancer, HP = *Helicobacter pylori*, IQR = interquartile range, L = lower third of the stomach, M = middle third of the stomach, PMH = past medical history, SMD = submucosal or deeper invasion, U = upper third of the stomach, undiff. = undifferentiated adenocarcinoma.

**Table 3:** Multivariate analysis to evaluate the 5 background factors based on the presence of submucosal invasion of gastric cancer.

	Crude odds ratio (95% CI)	Adjusted odds ratio (95% CI)	*P*-value
Age	0.99 (0.95–1.03)	0.98 (0.94–1.03)	.55
Male sex	0.85 (0.43–1.70)	0.72 (0.34–1.50)	.38
Open type atrophy	1.68 (0.65–4.34)	2.31 (0.85–6.31)	.10
PMH of GC	0.57 (0.24–1.39)	0.65 (0.26–1.65)	.37
L10AE	2.21 (1.08–4.52)	2.43 (1.15–5.11)	.019

CI = confidence interval, GC = gastric cancer, L10AE = longer than 10 years after eradication, PMH = past medical history.

### 3.3. Male sex, longer SI, and closed type gastritis are associated with GC developed at longer than 10 years after *H pylori* eradication

**Figure 2.**
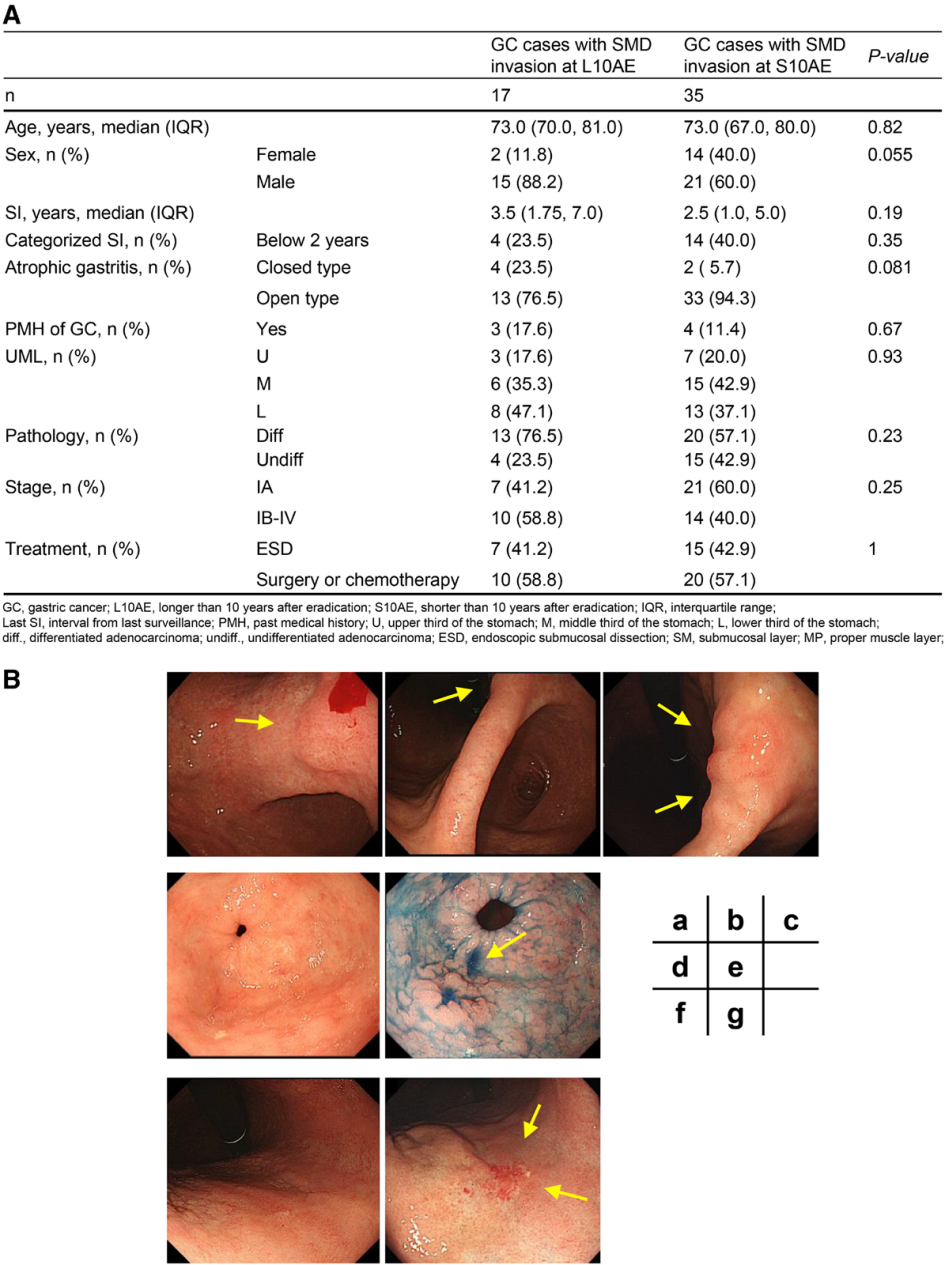
(A) Clinical and pathological characteristics of GC cases with SMD invasion developed at L10AE (n = 17) and S10AE (n = 35). Patients with GC diagnosed at L10AE tended to be male, with longer SI, and have associated closed atrophic gastritis. There were no obvious differences in pathological status and stage of tumor between the 2 groups. (B) GC cases at L10AE that had difficulty in recognizing the lesions on previous images within 2 years: Lesion I (a, b, c): the elevated lesion was identified on imaging 3 years before diagnosis, but the biopsy result was group 1 and was not recognized 1 year earlier. ESD was performed and the tumor showed a 150 μm depth of submucosal invasion. Lesion II (d, e): at 2 years before diagnosis, the lesion was not recognized (d). During diagnosis, it was recognized as a depressed lesion with a spiculated extension, and biopsy showed well-differentiated and moderately differentiated adenocarcinoma (e). ESD was performed and showed an invasive carcinoma with 750 μm submucosal invasion. Lymphatic invasion is detected with a D2-40 stain. Lesion III (f, g): granular mucosa is recognized on the anterior wall of the lower body with small erosions but was group 1 on biopsy (f). After 1 year, the lesion is clearly recognizable as a whitish depressed lesion (g). A reddish insular regenerated mucosa is observed at the center of the depression. ESD and additional operations were performed, resulting in a diagnosis of tubular adenocarcinoma (tub2 > por), M, Less-Ant, 0-IIc, 45 × 51 mm, pT1b2 (SM2, 1000 μm), Ly1, v1, HM0, VM0, N1. Ant = anterior; GC = gastric cancer; ESD = endoscopic submucosal dissection; L10AE = longer than 10 years after eradication of *H pylori*; Less = lesser curvature; Ly = lymph vessel invasion; M = middle third of the stomach; N = regional lymph nodes; poor = poorly differentiated adenocarcinoma; S10AE = shorter than 10 years after eradication of *H pylori*; SI = surveillance interval; SM = submucosa; SMD = submucosal or deeper invasion; tub2 = moderately differentiated adenocarcinoma; V = venous invasion; VM = vertical margin.

Finally, the 52 GC patients with SMD invasion were categorized into 2 groups according to the period from eradication of *H pylori* (Fig. 1). Regarding age and past medical history of GC, there were no significant differences between the 17 GC cases at L10AE and 35 GC cases at S10AE (Fig. 2A). Though not significant, male sex, longer SI, and closed type atrophic gastritis tended to be associated with GC cases at L10AE (Fig. 2A). There were no obvious differences in pathological status and stage of tumor between the 2 groups. Of the 17 cases of invasive GC developed at L10AE, 4 cases had SIs of <2 years. Endoscopic images of 3 of these 4 cases are shown in Figure 2B. These GC cases illustrate the difficulty in recognizing the tumor lesions during GC development at L10AE in comparison with S10AE.

## 4. Discussion

In this study, we examined the associated factors and clinicopathological characteristics of GC developed at L10AE in comparison with those developed at S10AE. Interestingly, we identified 2 characteristics of GC developed at L10AE. First, SI was a significantly associated factor for the development of GC at L10AE. Second, GC with SMD invasion was significantly more frequently observed in L10AE cases.

No studies have directly investigated the risk factors for GC developed at L10AE, and no obvious risk factors have been noted. Therefore, this is the first study showing that SI is an important risk factor. Concerning invasive GC after eradication of *H pylori*, Kobayashi et al pointed out that annual endoscopy is associated with earlier cancer detection.^[16]^ Additionally, data from South Korea’s cancer screening program showed that more frequent endoscopic screenings are associated with a reduced risk of GC mortality.^[17]^ In addition to the need for continuous endoscopic surveillance demonstrated in a previous report,^[7]^ it is important to note that the occurrence of GC with SMD invasion was more frequently detected in L10AE than in S10AE cases. We believe that detecting intramucosal GC lesion is clinically important, because almost all of such lesions can be resected by endoscopy. In our study design, we analyzed GC cases which were detected in the screening endoscopy. To be precise, our study design cannot distinguish the change of GC development/occurrence from the delayed detection of GC. However, whether it is the change of development/occurrence or the detection of GC, it is important to diagnose GC in earlier stages from the clinical aspect. Our result clearly showed that SI is a crucial factor for strategy against GC even at L10AE.

The identification of SI as a factor related to GC development in L10AE suggests the involvement of the following characteristics. Concerning differentiated GC cases, the demarcation line of the lesions may be unrecognizable owing to gastritis-like appearance,^[18]^ or the presence of the epithelium with low-grade atypia in the GC surface layer.^[19]^ Eradication of *H pylori* may flatten the lesions, also making it difficult to recognize them.^[20]^ Among these, the gastritis-like appearance tends to appear more frequently in patients with GC developed at L10AE compared with those developed at S10AE.^[21]^ These factors may result in failure to recognize GC developed at L10AE.^[22]^

SI should not be extended in patients with GC development at L10AE when the appropriate SI after eradication, and number of years for which it should be continued, have not been defined. Kobayashi et al identified examination intervals as a factor for invasive cancer even after 10 years post-eradication.^[16]^ Our study, including intramucosal carcinoma, also demonstrated similar findings. The number of patients in this study was too small for a conclusion based on the evidence. However, our results suggested that SI should not be extended in these patients. In the future, it is necessary to establish an appropriate SI for patients with GC development at L10AE using a large-scale prospective design study.

The effectiveness of eradication therapy in healthy individuals without a history of GC only represented approximately 66% of the risk of developing GC.^[23]^ It remains unknown whether SI should be shortened for all patients to prevent post-eradication invasive GC and rapidly developing cancers. Furthermore, risk stratification is desirable and future research in this area is anticipated.

Our study has some limitations. First, this was a single-center retrospective study. The small number of patients limited the power of the statistical methods and affected the power of the analysis. Second, pathological diagnosis and evaluation were performed at a single-center without a central review of diagnostic pathology. Third, there is a potential selection bias, where patients with longer follow-up periods may have longer SIs. Fourth, our study design cannot distinguish the change of GC development/occurrence from the delayed detection of GC. However, whichever the case may be, early diagnosis of GC is important, as described above.

This study provides important recommendations for avoiding development of GC with SMD invasion developed at L10AE. GC is still observed with a longer SI, and GC with SMD invasion is more frequently detected in L10AE than in S10AE. Even if the SI was less than 2 years, there were some cases of invasive GC developed at L10AE. It is suggested to avoid extending SI or terminating follow-up at more than 10 years after eradication. Further research is needed to determine the optimal SIs.

## Acknowledgments

The authors thank Editage (www.editage.com) for English language editing.

## Author contributions

**Conceptualization:** Akiko Sasaki.

**Data curation:** Akiko Sasaki.

**Formal analysis:** Akiko Sasaki, Chikamasa Ichita.

**Investigation:** Akiko Sasaki, Chihiro Sumida, Takashi Nishino, Miki Nagayama, Jun Kawachi, Yuma Suno, Takaaki Murata, Wataru Naito.

**Methodology:** Akiko Sasaki, Chikamasa Ichita.

**Project administration:** Akiko Sasaki.

**Resources:** Akiko Sasaki.

**Supervision:** Nobutake Yamamichi.

**Visualization:** Akiko Sasaki.

**Writing – original draft:** Akiko Sasaki.

**Writing – review & editing:** Akiko Sasaki, Nobutake Yamamichi.
